# Photoactivation of [FeFe] Hydrogenase Studied by Multiscale Time-Resolved Infrared Spectroscopy

**DOI:** 10.1021/acs.jpclett.6c00408

**Published:** 2026-03-27

**Authors:** Elizaveta Kobeleva, Malin Khalil, Manon T. Lachmann, Partha Malakar, Sayantan Bhattacharya, Gregory M. Greetham, Patricia Rodriguez-Maciá, James A. Birrell, Marius Horch

**Affiliations:** † Department of Physics, Ultrafast Dynamics in Catalysis, 9166 Freie Universität Berlin, Arnimallee 14, 14195 Berlin, Germany; ‡ School of Chemistry and Leicester Institute for Structural and Chemical Biology, 4488 University of Leicester, University Road, Leicester LE1 7RH, U.K.; § Research Complex at Harwell, Rutherford Appleton Laboratory, STFC Central Laser Facility, Harwell Campus, Didcot OX11 0QX, U.K.; ∥ School of Life Sciences, University of Essex, Wivenhoe Park, Colchester CO4 3SQ, U.K.

## Abstract

[FeFe] hydrogenases catalyze the reversible cleavage of H_2_, a clean fuel, at exceptional rates. Therefore, understanding their catalytic mechanism is of high importance. Here, we employ multiscale UV_pump_-IR_probe_ spectroscopy to study the reversible photochemical activation of the CO-inhibited H_ox_-CO state over picosecond to millisecond time scales. Since this process transforms the catalytic site into the active, H_2_-binding H_ox_ state, photolysis of H_ox_-CO represents a unique strategy for studying light-triggered activation and the catalytic cycle of the enzyme with high time resolution. We show that the extrinsic CO of H_ox_-CO dissociates in picoseconds and remains unbound for up to milliseconds. During this time, the enzyme is available for H_2_ binding and further catalytic transformations. This time window is sufficiently large to study catalytic processes from the earliest steps to completion of the catalytic cycle. Our approach provides a basis for investigating the catalytic cycle of [FeFe] hydrogenases in real time and without diffusion limitation.

## Graphical abstract

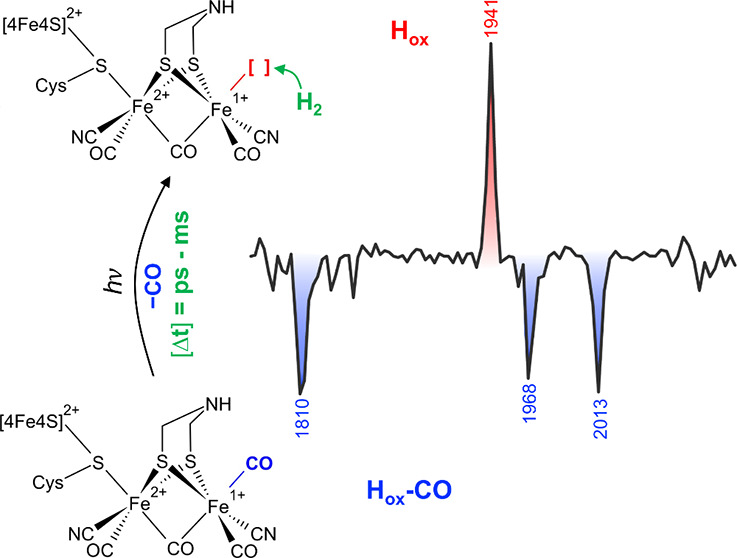

Hydrogenases are metalloenzymes catalyzing the reversible cleavage of H_2_, which has gained a lot of attention for its potential utilization as a clean and sustainable fuel.^1^ [FeFe] hydrogenases form the most efficient subclass of hydrogenases.^2–4^ Their active site, typically called the H-cluster, consists of a binuclear [2Fe]_H_ subsite and a [4Fe-4S]_H_ subcluster, connected via a cysteine ligand (Figure 1).^5,6^ The [2Fe]_H_ subsite features a total of five intrinsic diatomic ligands, three CO and two CN^–^.^7,8^ In addition, the two Fe ions, called Fe_p_ (proximal to the [4Fe-4S]_H_ cluster) and Fe_d_ (distal to the [4Fe-4S]_H_ cluster), are bridged by a 2-azapropane-1,3-dithiolate (ADT) ligand. This ligand is involved in proton transfer and the heterolytic cleavage of H_2_ by a frustrated Lewis pair made of the aza group and a vacant coordination site on Fe_d_.^9^ In the CO-inhibited state of [FeFe] hydrogenase, this vacant coordination site is occupied by an extrinsic CO ligand (Figure 1), which prevents catalytic activity.^10–12^

**Figure 1:**
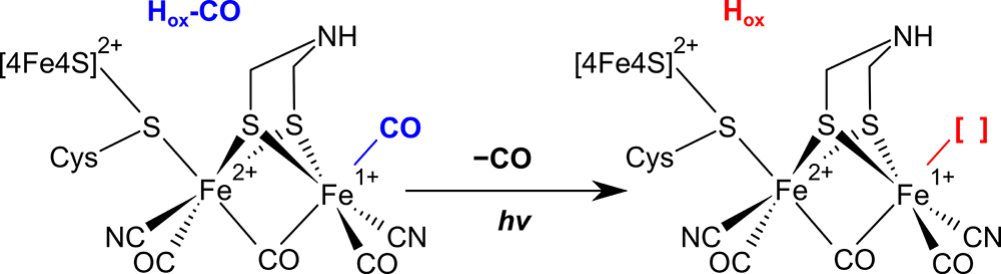
Structure of the active site of [FeFe] hydrogenase in the CO-inhibited H_ox_-CO state, where the photolyzable CO ligand is shown in blue. The [2Fe]_H_ subsite is shown explicitly, while the [4Fe-4S]_H_ subcluster is indicated by its molecular formula.

Despite extensive research, the catalytic mechanism of [FeFe] hydrogenases is not fully understood. One of the key limitations is a lack of real-time insights and experimental access to the earliest catalytic intermediates of H_2_ cleavage, the structures of which were so far only postulated but not observed.^9^ Consequently, rate constants of associated early steps are not known, so that investigations over various time scales are necessary. Besides that, hydrogenases are inherently difficult to study with high time resolution, as they are ground-state catalysts. As a consequence, their catalytic reaction *as a whole* cannot be easily triggered by light, which is the only option to access short time scales. This is a general problem in the investigation of fast ground-state catalysts, highlighting the necessity to develop adequate strategies to trigger such systems with light. In addition, the earliest catalytic steps of H_2_ oxidation are preceded by solvent diffusion and intramolecular mass transport (of H_2_). If these processes are slow relative to the initial chemical steps, the associated intermediates cannot be observed in an ensemble experiment irrespective of the instrumental time resolution. However, it has been shown that many active and inactive states of hydrogenases are light-sensitive, allowing for triggering various *individual* catalytic steps with light,^11,13,14^ thereby allowing studying them with high time resolution and unaffected by mass transport limitation.

Here, we utilize this approach by combining UV/vis excitation (actinic pump) with IR detection (probe) in a UV/vis_pump_-IR_probe_ spectroscopic study. IR spectroscopy has proven to be a powerful technique for investigating all redox-structural states of hydrogenases, due to the IR-active and structurally sensitive bond-stretching vibrations of the CO and CN^–^ ligands at their active sites.^7–9^ To cover picosecond to millisecond time scales in a single time-resolved (TR) experiment, we use multiscale UV/vis_pump_-IR_probe_ spectroscopy with a pump-multiprobe pulse delay scheme.^15–17^ In this method, an ultrashort femtosecond pump pulse in the UV to visible range is followed by a set of IR probe pulses, centered on the region of the CO and CN stretching vibrations, 1750–2150 cm^–1^. The electronic transitions of the [2Fe]_H_ subsite are not known, due to domination of the absorption spectrum by the [4Fe-4S]_H_ subcluster. For this work, we choose UV pump excitation at 350 nm, which gave the highest signal intensities among tested excitation wavelengths (others were 460 and 640 nm). Varying the time delay between pump and probe pulses allows following reaction dynamics over all relevant time scales after light-triggered initiation of a process of interest.

Here, we focus on the oxidized CO-inhibited state (H_ox_-CO) of the [FeFe] hydrogenase from *Chlamydomonas reinhardtii* (*Cr*HydA1). Illumination of the inactive and oxygen-protected H_ox_-CO state results in photolysis of the extrinsic CO ligand,^11,18^ thereby forming the most oxidized catalytic intermediate, H_ox_, which is generally accepted as the H_2_-binding state of [FeFe] hydrogenase.^9,10^ Therefore, photolysis of H_ox_-CO in combination with multiscale TR-IR spectroscopy represents a unique strategy for studying the light-triggered activation of the enzyme and all associated and subsequent processes in real time. Of note, all involved states can be distinguished by IR spectroscopy through the characteristic frequencies of the CO and CN stretching modes. These frequencies are explicitly known for H_ox_ (1800, 1941, 1964, 2072, and 2088 cm^–1^) and H_ox_-CO (1810, 1967, 1970, 2013, 2084, and 2092 cm^–1^).^18^

While rebinding of the CO ligand on millisecond time scales was studied previously,^19^ the ultrafast photolysis of the CO ligand itself is so-far unexplored. Here we use ultrashort excitation pulses to follow and analyze CO dissociation and rebinding as well as vibrational energy dissipation dynamics in real time and under ambient conditions. Notably, by rapidly forming the H_2_-binding intermediate H_ox_, photolysis of H_ox_-CO represents an entry point to the catalytic cycle. Thus, in the presence of saturating H_2_ concentrations, the complete catalytic cycle could be explored, over multiple time scales and without mass transport limitation. In particular, this would provide access to the earliest catalytic steps and intermediates of [FeFe] hydrogenases, which are key to understanding biological H_2_ activation. To lay a foundation for such experiments, we concentrate here on the initial step of CO photodissociation to explore the time scales of CO photolysis and thermal rebinding to define the accessible time window for H_2_ binding in such experiments.

In the pump-multiprobe experiment, an ultrashort UV pump pulse excites the sample and promotes the system from its ground state to an electronically excited state. The subsequent time evolution of the system is then followed by a set of IR probe pulses, and the data are represented as a set of time-dependent difference spectra. Upon excitation, the original ground-state population decreases, which gives rise to negative signals at the fundamental IR absorption frequencies (0–1) of the bleached states. The corresponding photoinduced states appeared as positive signals. Pump–probe spectra were recorded sequentially covering picosecond, nanosecond, and microsecond time scales. For clarity, data from those time scales, reflecting different physical and chemical processes, will be discussed separately.

On early picosecond time scales, pump–probe spectra consist of intense bleach signals and less intense positive signals (Figure 2A,B). Frequencies of all dominant negative signals correspond to fundamental (0–1) transitions of the CO stretch modes (1810, 1967, 1970, and 2013 cm^–1^) and CN stretch modes (2084 and 2092 cm^–1^) of the H_ox_-CO state.^18,20,21^ Of note, dominant CO stretch signals at 1967 and 1970 cm^–1^ are not fully resolved and appear as one overlapping peak with a joint maximum at 1968 cm^–1^. Additional minor signals in the CO stretch region (1941 and 1947 cm^–1^) exhibit very short lifetimes (ca. 3 ps; fits are shown in Figure S1D), which indicates that they represent a contamination of some sort, e.g. water-exposed H-cluster degradation products,^22^ rather than any known state of [FeFe] hydrogenase (e.g., H_ox_ and H_ox_H). Therefore, we do not discuss them in the following. In the region of the CO stretching vibrations, positive signals are also observed at lower frequencies. The apparent displacement between positive and negative signals are similar to typical anharmonicities of the CO stretching modes (14–30 cm^–1^).^23^ This indicates that the positive signals correspond to the same state, H_ox_-CO, and reflect transitions between the first and second vibrationally excited states of the individual CO stretch modes (1–2).

**Figure 2:**
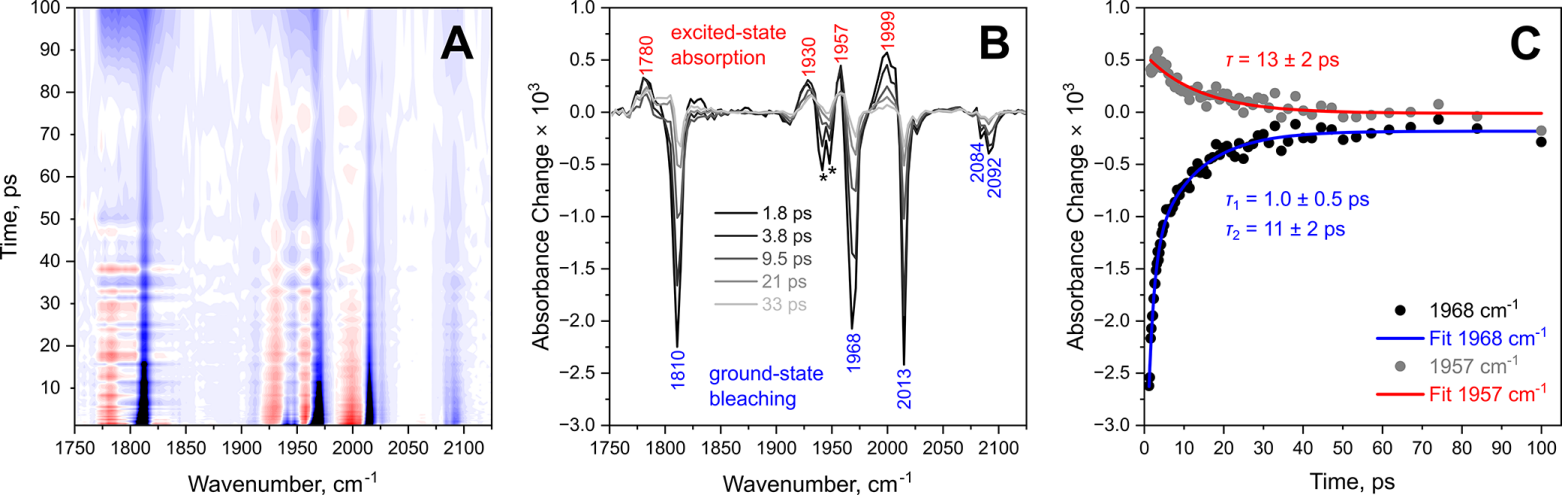
UV_pump_-IR_probe_ spectra of *Cr*HydA1 poised in the H_ox_-CO state, recorded with picosecond delays and extracted kinetics. Data were recorded up to 500 ps, but no significant changes were observed after 100 ps. (A) Contour plot from 1 to 100 ps. Negative signals are shown in blue, and positive signals are shown in red. (B) Selected pump–probe spectra. (C) Time traces of the apparent maxima of selected signals with time constants extracted by exponential or biexponential fits.

To explore processes on early picosecond time scales in more detail, the time evolution of the observed signals was analyzed by following the signal intensity at the position of the apparent maximum for each peak. Representative kinetic traces and associated fits are shown in Figure 2C, and more detailed information can be found in Figure S1 and Table S1.

The time evolution of the negative signals corresponding to the parent state (electronic and vibrational ground states of H_ox_-CO) is the same for all signals and varies only within the error margin. It exhibits a biexponential decay with time constants of 1 ± 0.5 and 11 ± 2 ps, indicating two independent processes. Positive signals also exhibit a common time evolution but decay monoexponentially with a time constant of 13 ± 2 ps, which match the slower process of the negative signals. Based on the frequency positions and the time evolution of the signals, we propose the following simplified mechanism: After electronic excitation, the H-cluster in the H_ox_-CO state returns to the electronic ground state by ultrafast internal conversion within the response time of the instrument (200–300 fs). This process yields a hot electronic ground state with the energy from electronic excitation distributed across multiple degrees of freedom. Specifically, a significant portion of energy (about one-quarter) ends up in the CO stretch modes, as indicated by positive signals associated with 1–2 transitions that are observed from earliest accessible delay times on. These signals decay due to vibrational relaxation with a time constant that matches the slower decay component of the negative bleach signals (11 ± 2 ps) and the expected vibrational lifetimes of the CO stretch modes. These observations indicate a peculiar relaxation mechanism resulting in directed energy transfer toward the CO stretching modes. In addition, the biexponential recovery of the parent state, as observed through the bleach-signal decay, suggests a second dominant pathway of vibrational relaxation (about three-quarters of the overall energy). Since the corresponding small time constant of 1 ± 0.5 ps is not observed for the positive signals, we assign this process to ultrafast vibrational cooling via a bath of vibrational modes. In this case, energy dissipation occurs in a typical delocalized manner via various vibrational modes and not through a single vibrational coordinate. Therefore, the CO stretch mode is not shifted by a single well-defined anharmonicity. Instead, a continuum of transition energies (positive signals) is observed, which leads to a broad and rapidly decaying positive offset in early picoseconds spectra. This effect cannot be separated from general baseline drift, which is subtracted from all data shown in the manuscript.

Vibrational dynamics are clearly completed at 100 ps, and about 90% of the original parent state population is recovered by this time. However, about 10% of the bleach-signal intensity remains, as can be deduced from an offset in the exponential decay models and the original amplitude at time zero (obtained from the fit). This demonstrates that not all molecules have returned to the overall ground state of the H_ox_-CO species by the processes discussed above. Consequently, this fraction is available for photochemical processes, including the photolysis of the H_ox_-CO state. This calculation also yields an upper limit for the CO-photolysis quantum yield in the current experimental settings.

Part of the electronically excited molecules do not return to the initial state via ultrafast internal conversion and vibrational cooling but undergo photochemical conversion. Specifically, the external CO ligand of H_ox_-CO gets photolyzed and the H_ox_ state is formed. Spectroscopically, this can be demonstrated via the appearance of positive signals corresponding to the CO/CN stretching vibrations of the H_ox_ state, the most pronounced of which appears at 1941 cm^–1^, well separated from any H_ox_-CO 0–1 transition. However, direct detection of this signal is still complicated at early times by overlap with more intense signals of impurities and 1–2 transitions of H_ox_-CO occurring in the same frequency range. Therefore, the time constant of photochemical H_ox_ formation is difficult to define. Nevertheless, well-resolved spectroscopic signatures of this state are observed from late picosecond to nanosecond time scales after vibrational relaxation is completed, so that signatures of the hot electronic ground state do not overlap with those of H_ox_ (Figure 3).

**Figure 3:**
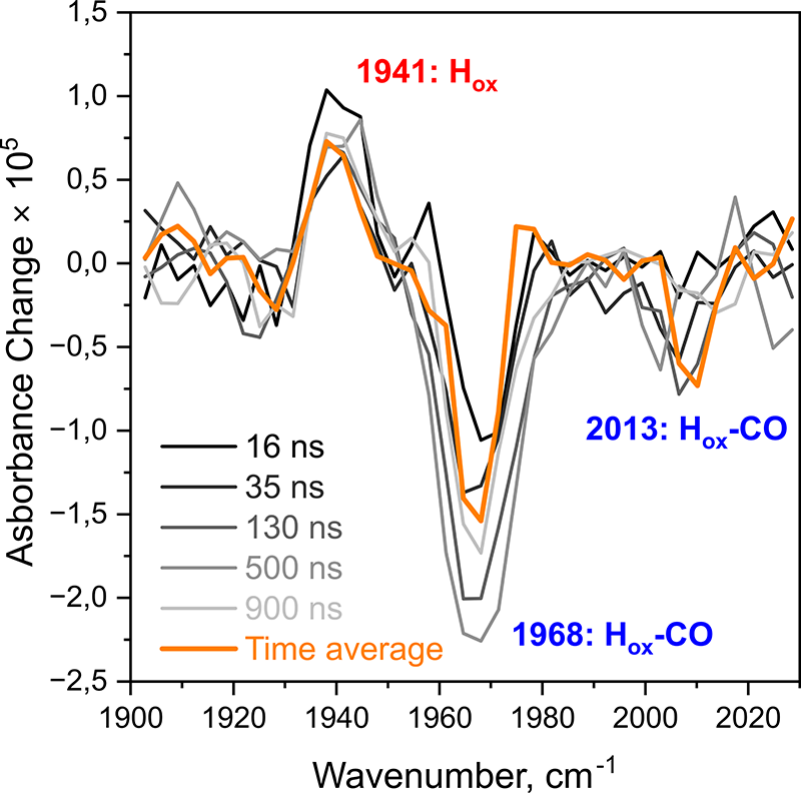
UV_pump_-IR_probe_ spectra of *Cr*HydA1 poised in the H_ox_-CO state, recorded with nanoseconds delays. Individual spectra were selected from data recorded between 1 and 1000 ns (also see Figure S2). In addition, a spectrum averaged over the entire nanosecond time range is shown in orange. Spectra were smoothed by using a Savitzky–Golay filter. Intensities are not directly comparable to picosecond and microsecond data due to different sample concentrations and pump conditions.

Following pump–probe spectra recorded at nanosecond delays confirms the formation of H_ox_ (Figure 3, Figure S2). Interestingly, throughout the entire nanosecond range, no significant population change of the product state, H_ox_, or the parent state, H_ox_-CO, is detected. As shown in Figure S2, both states, associated with positive and negative peaks at 1941 and 1967 cm^–1^, respectively, exhibit constant kinetic traces over the whole nanosecond range. All intensity variations are within the error margin and do not show any observable trend. This observation confirms that the photoreaction occurred before those times and that the product is already fully formed during picosecond time scales. Even though we could not reliably determine the photoproduct formation time constant from our data, this finding allows estimation of an upper limit of hundreds of picoseconds. Consistently, various reports^24–28^ on [FeFe] model complexes report even faster CO-photolysis rates, on the order of few picoseconds, supporting ultrafast ligand dissociation. In addition, the constant intensities of H_ox_ and H_ox_-CO marker bands suggests that the photoinduced H_ox_ state remains stable at least over nanosecond time scales.

Pump–probe spectra at microsecond delays also reveal clear positive signals corresponding to the H_ox_ state and negative signals corresponding to the H_ox_-CO state (Figure 4, Figure S3). Early microsecond time scales show a continued growth of positive signals, indicating a slow secondary process leading to the formation of H_ox_. Overall, the kinetic trace of H_ox_ can be modeled with a biexponential function. Here, the growth component is characterized by a time constant of 17 ± 2 μs and the decay component by a time constant >1 ms, which is beyond the measurement time frame. The observation of two processes for H_ox_ photoformation, one on picosecond time scales and one on microsecond time scales, could indicate two pathways of CO dissociation. While picosecond CO photolysis is expected from work on [FeFe] model complexes and other iron carbonyl species,^24–26,29–32^ the slower CO photolysis is without precedence, to the best of our knowledge. The absence of an associated loss of the H_ox_-CO parent state, characterized by a similar time constant, suggests that the slower formation of the H_ox_ photoproduct occurs indirectly via an intermediate state. Since no spectroscopic signature of the intermediate state can be identified, we suggest that it is structurally similar to and spectroscopically indistinguishable from the H_ox_-CO and/or H_ox_ states, likely excluding a significant rearrangement in the inner coordination sphere of the H-cluster. This interpretation is consistent with the observation that relative intensities of signals assigned to the H_ox_-CO state change between time scales (see Figures 2B, 3 and 4B). Since the process is orders of magnitude slower than the nonradiative decay of the initial electronically excited state (picoseconds time scales), we furthermore suggest a thermally activated ground-state step in the formation of H_ox_. A possible scenario would be the photochemical formation of an intermediary state that is thermodynamically unstable and separated from H_ox_ by a smaller reaction barrier than from H_ox_-CO, thereby preventing direct relaxation to the latter. Such an intermediate could be transformed to the canonical H_ox_ state via a thermal process characterized by an observed formation time constant of 17 ± 2 μs.

**Figure 4:**
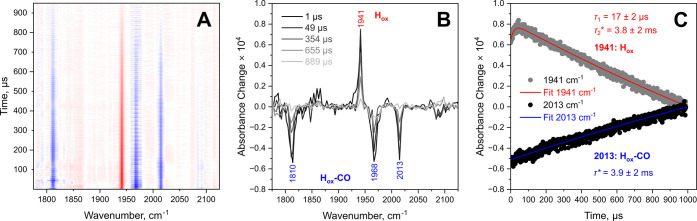
UV_pump_-IR_probe_ spectra of *Cr*HydA1 poised in the H_ox_-CO state, recorded with microsecond delays (1 to 989 μs) and extracted kinetics. (A) Contour plot from 1 to 950 μs. (B) Selected pump–probe spectra. (C) Time traces of the apparent maxima of selected representative signals with time constants extracted by exponential or biexponential fits. Apparent time constants are marked with an asterisk.

At longer microsecond time scales, the photoinduced H_ox_ state exhibits slow exponential decay, accompanied by a slow exponential recovery of the H_ox_-CO parent state, reflecting the CO rebinding process. Both processes are characterized by apparent time constants >1 ms (see Table S2) and therefore visually linear in appearance over the time range probed. Of note, these time constants are beyond the measurement time frame and therefore less reliable than the other determined quantities. In principle, their values might also be affected by sample refreshment, i.e., movement of the pumped sample out of the probe beam before relaxation is complete. This would explain why the time traces decay to zero at 1 ms, and the apparent time constants would represent a lower limit of the real ones. In fact, millisecond decay of the photoinduced state and recovery of the parent state are in line with previous studies and the somewhat larger time constant determined there (13 ms).^19^ Therefore, this observation demonstrates that the photochemically formed H_ox_ state is stable over at least 8 orders of magnitude in time, from ≤10^–10^ to ≥10^–3^ s, and proves the reversibility of CO dissociation. Together these two aspects form a basis for investigating the catalytic cycle of [FeFe] hydrogenases by this method: After CO photodissociation, the activated enzyme can readily bind H_2_, leading to the formation of various catalytic intermediates and launching the entire catalytic cycle. Of note, the shortest time scales of CO photolysis approach the upper limit of the estimated rate of the first catalytic step of H_2_ cleavage,^33^ while the longest time scales exceed the duration of a full catalytic cycle, as judged by H_2_ turnover frequencies of [FeFe] hydrogenases.^2,4,34^ As such, the introduced approach promises access to the entire catalytic cycle, including the earliest elementary steps of H_2_ binding and activation.

In total, our work demonstrates the benefits of using multiscale UV_pump_-IR_probe_ spectroscopy to study photophysical and photochemical processes in [FeFe] hydrogenases. At the earliest picosecond time scales, vibrational dynamics following ultrafast internal conversion was studied, and two pathways of energy dissipation were discovered: directional via selected CO stretching modes (ca. 11 ps) and delocalized via a bath of vibrational modes (ca. 1 ps). In addition, CO photolysis from the H_ox_-CO state, yielding H_ox_, was characterized. We showed that the extrinsic CO of H_ox_-CO dissociates on picosecond time scales and remains unbound up to milliseconds. Besides that, an additional photoinduced intermediate appears to transform thermally to H_ox_ on the early microsecond time scale.

Fast CO dissociation and slow reversible CO rebinding provide a periodic time window of at least 8 orders of magnitude, from ≤10^–10^ to ≥10^–3^ s, during which the enzyme is in its active form and available for interaction with H_2_ and further catalytic transformations. This provides a perfect framework for studying H_2_ binding and conversion as the long time window allows exploring catalytic processes and associated intermediates from the earliest steps (estimated ≤10^–11^ s)^33^ to the completion of the cycle (on the order of 10^–5^ s). The clear kinetic picture and sufficient level of photoconversion serve as proof of concept and form a basis for further investigation of catalytic intermediates of hydrogenases and, subsequently, its catalytic mechanism with the given experimental method.

In a wider sense, the combination of multiscale time-resolved IR spectroscopy with the photoactivation of reversibly inhibited (bio)­catalysts by ultrashort light pulses provides new perspectives for understanding the mechanism of enzymes and other fast ground-state catalysts.

## Experimental Procedures

The apo-[FeFe] hydrogenase from *Cr*HydA1 was recombinantly expressed in *E. coli* and matured with the [2Fe]^ADT^ cofactor as described previously.^4,35^ The CO-inhibited H_ox_-CO state was prepared by flushing enzyme in the H_ox_ state with 100% CO gas for 15 min.^36^ Samples were concentrated to approximately 3 mM in 100 mM Tris/HCl and 150 mM NaCl buffer at pH 8.

Time-resolved UV_pump_-IR_probe_ spectra were recorded in time-resolved multiprobe spectroscopy (TR^M^PS) mode using the LIFEtime laser system of the STFC Central Laser Facility. A detailed description of the setup and the methodology can be found in references 15–17 . Briefly, a 100 kHz ultrafast laser based on a custom dual Yb:KGW system (Pharos, Light Conversion) pumped pump and probe optical parametric amplifier (OPA) used for UV and MIR generation. These OPAs provided broadband MIR probe pulses (ca. 200 cm^–1^) centered at 1925 and 2070 cm^–1^ (0.05 μJ, ca. 200 fs) and 350 nm UV pump pulses (0.6 μJ, ca. 300 fs). The polarization of pump and probe pulses was set to the magic angle. The probe light was detected using two 128-pixel MCT detectors, yielding a resolution of 2–3 cm^–1^. The sample holder was rastered to avoid photodamage by the UV pump pulse. Samples were kept under anaerobic conditions at all times, using a gas-tight transmission cell with two CaF_2_ windows separated by a 50 μm Teflon spacer (ca. 10 μL sample volume). Experiments were performed at ambient temperature (21 °C).

The spectra were frequency-calibrated by using various known states of [FeFe] hydrogenases as a reference via polynomial fitting. Picosecond, nanosecond, and microsecond timeframes were recorded in separate experiments to adjust accumulation times in accordance with the signal intensities: 6 s per delay point for picosecond time scales, 20 s for nanosecond time scales, and 30 s for microsecond time scales. All shown spectra were baseline corrected via spline functions in Matlab.^37^ Nanosecond spectra were additionally filtered for better representation since signal intensities were significantly lowered due to decreased sample concentration and different pump conditions. For this, a Savitzky–Golay filter with a window size of 3 points, as implemented in Matlab, was used. All the time traces were fit with exponential or biexponential functions using the lsqcurvefit routine in Matlab.^37^

## Supplementary Material



## Notes

Additional time traces with fits for various time scales (Figures S1–S3) and time constants extracted from the fits (Tables S1 and S2) (PDF)
