# Supplementary material for: Photoactivation of [FeFe] Hydrogenase Studied by Multiscale Time-Resolved Infrared Spectroscopy
Source: J Phys Chem Lett. 2026 Mar 27;17(14):4214–20. doi: 10.1021/acs.jpclett.6c00408 (PMC13071907; doi:10.1021/acs.jpclett.6c00408)
Supplement: Supplementary file 1 [file jz6c00408_si_001.pdf]

Supplementary Information

Photoactivation of [FeFe] Hydrogenase Studied by Multiscale Time-Resolved Infrared Spectroscopy

Elizaveta Kobeleva[1], Malin Khalil[1], Manon T. Lachmann[2], Partha Malakar[3], Sayantan Bhattacharya[3], Gregory M. Greetham[3], Patricia Rodriguez-Maciá[2], James A. Birrell[4] and Marius Horch[1]*

[1] Freie Universität Berlin, Department of Physics, Ultrafast Dynamics in Catalysis, Arnimallee 14, 14195 Berlin, Germany. E-mail: marius.horch@fu-berlin.de

[2] University of Leicester, School of Chemistry and Leicester Institute for Structural and Chemical Biology, University Road, Leicester, LE1 7RH, UK

[2] STFC Central Laser Facility, Research Complex at Harwell, Rutherford Appleton Laboratory, Harwell Campus, Didcot, OX11 0QX, UK

[3] University of Essex, School of Life Sciences, Wivenhoe Park, Colchester, CO4 3SQ, UK

Contents

Supplementary Figures S1–S3 p. S1

Supplementary Tables S1–S2 p. S3

Supplementary Figures (all data correspond to *Cr*HydA1)

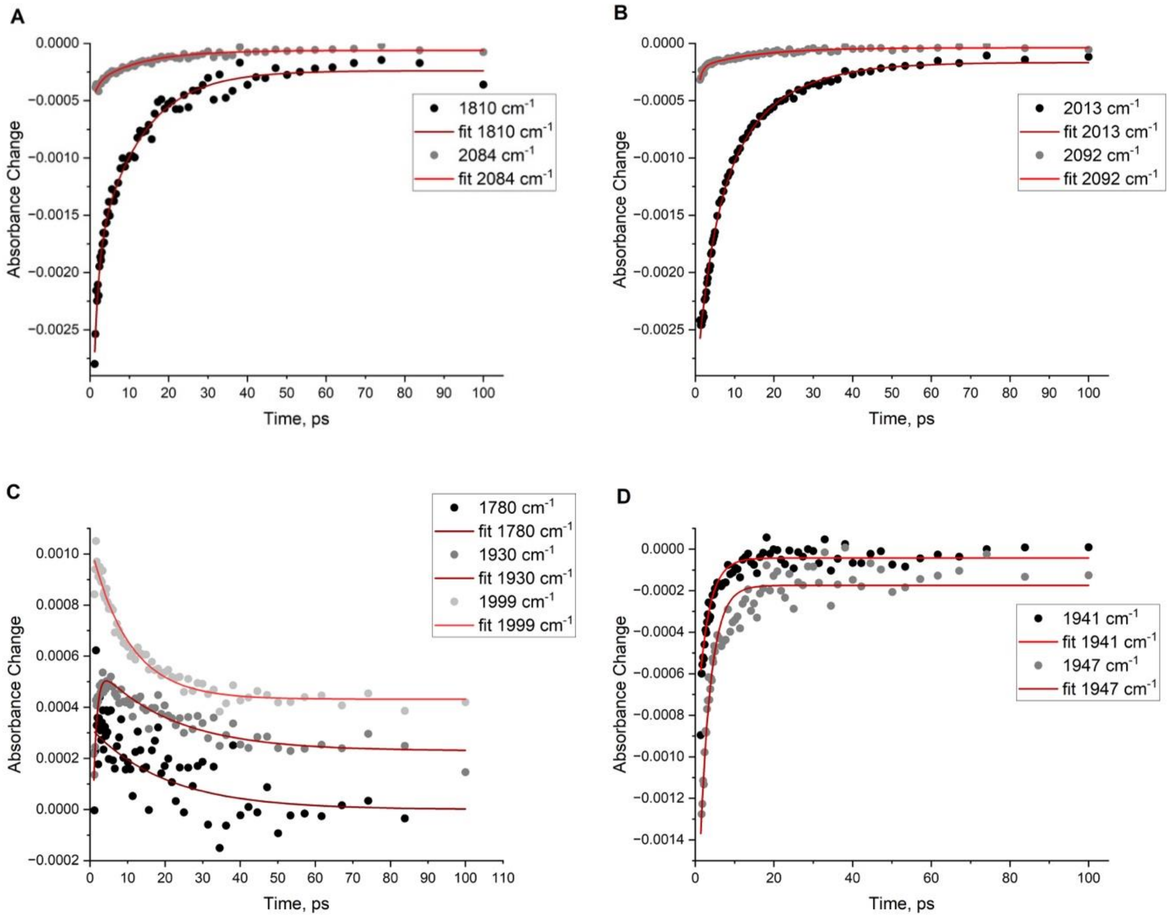

Figure S1: Time traces at picosecond timescales, obtained at indicated frequencies, reflecting apparent maxima of (A, B, D) negative signals and (C) positive signals. Signals in panels A–C reflect the H_{ox}-CO state, while those in panel D are from contaminant signals. The original data are shown as grey and black dots, while exponential and biexponential fits are shown as (dark) red lines. Positive traces in Figure S1C are plotted with a vertical offset for clearer representation. Extracted time constants are summarized in Table S1. The data correspond to those shown in Figure 2 of the manuscript.

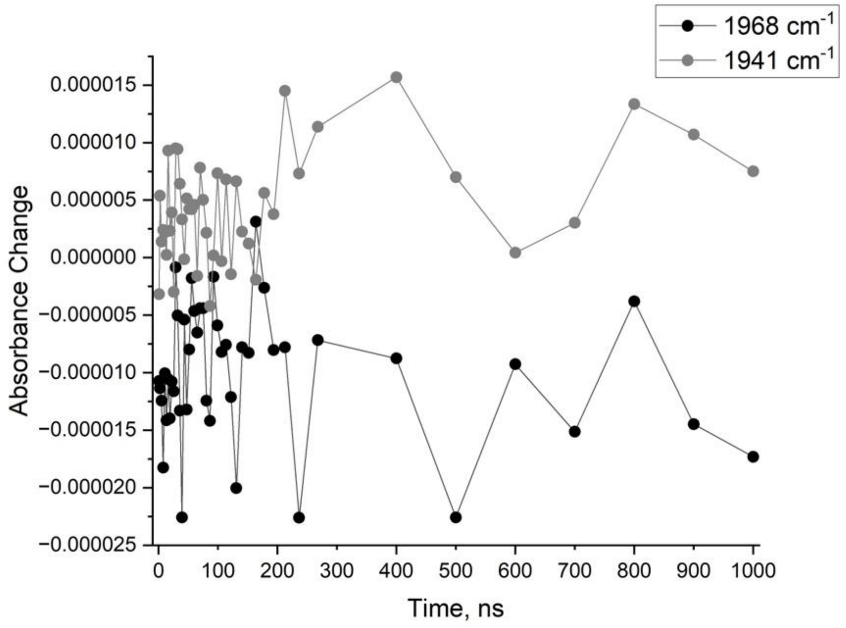

Figure S2: Time traces at nanosecond timescales, obtained at indicated frequencies representing the apparent maxima of signals that reflect the parent state H_{ox}-CO (1968 cm^{-1}) and the product state H_{ox} (1941 cm^{-1}) of the photolysis reaction. No systematic trend can be observed for the intensity of either signal, indicating *quasi*-static populations of both states on the probed timescales. The data correspond to those shown in Figure 3 of the manuscript.

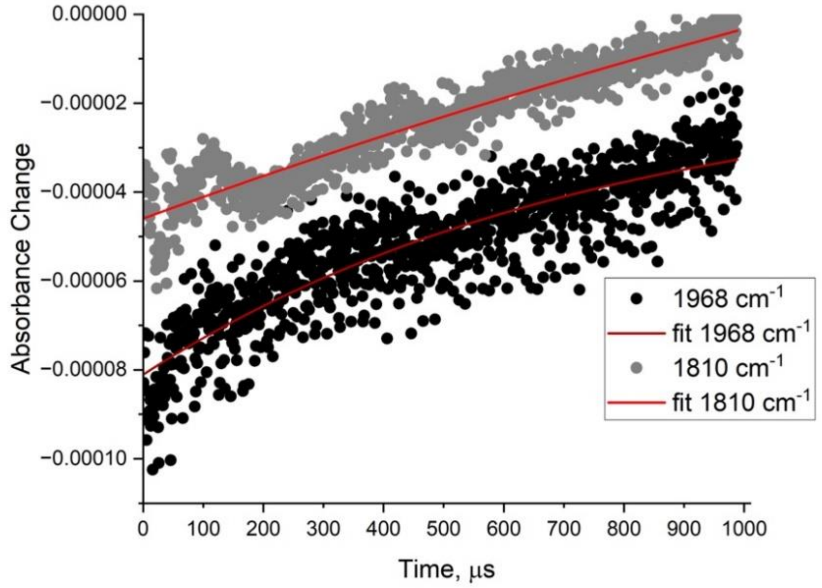

Figure S3: Time traces at microsecond timescales, obtained at indicated frequencies representing the apparent maxima of negative signals. The original data are shown as grey and black dots, while exponential and biexponential fits are shown as (dark) red lines. Time traces are plotted with a vertical offset for clearer representation. Extracted time constants are summarized in Table S2. The data correspond to those shown in Figure 4 of the manuscript.

Supplementary Tables (all data correspond to *Cr*HydA1)

Table S1: Time constants from exponential and biexponential fits to time traces obtained at the apparent maxima of peaks observed at picoseconds timescale (see Figure 2 and Figure S1). All transitions correspond to the H_{ox}-CO state, except for those marked as contaminants. Unless indicated otherwise, all time constants are decay constants.‡

Transition	Wavenumber, cm^{-1}	Time constant, ps	Time constant, ps
ESA μCO	1780	20±15	-
GSB μCO	1810	11±2	1.0±0.5
ESA tCO	1930	20±7	1.0±0.3**
GSB contaminant	1941	2.8±1	-
GSB contaminant	1947	2.7±1	-
ESA tCO	1957	13±2	-
GSB tCO*	1968	11±2	1.0±0.5
ESA tCO_{ext}	1999	10±1	-
GSB tCO_{ext}	2013	13±4	4±2
GSB CN1	2084	14±3	1±0.4
GSB CN2	2092	14±3	1.5±1

‡ESA, excited-state absorption; GSB, ground-state bleaching; μCO, bridging CO stretch mode; tCO, terminal CO stretch mode; tCO_{ext}, terminal CO stretch mode supposedly dominated by the bond stretching coordinate of the external CO.

*The apparent maximum corresponds to two unresolved transitions at ca. 1964 and 1970 cm^{-1}.

**The apparent growth component of the ESA signal is likely due to overlap with a nearby GSB peak reflecting impurities discussed in the main text.

Table S2: Time constants from exponential and biexponential fits to time traces obtained at the apparent maxima of peaks observed at microsecond timescales (see Figure 4 and Figure S3). Unless indicated otherwise, all transitions correspond to the H_{ox}-CO state, and all time constants are decay constants.‡

Mode	Wavenumber, cm^{-1}	Time constant, ms
GSB μCO*	1810	3±2
GSB tCO*	1968	0.8±0.2
GSB tCO_{ext}	2013	3.9±2
GSA tCO (H_{ox} – product)	1941	0.017±0.002**; 3.8±2

‡GSB, ground-state bleaching; GSA, ground-state absorption; μCO, bridging CO stretch mode; tCO, terminal CO stretch mode; tCO_{ext}, terminal CO stretch mode supposedly dominated by the bond stretching coordinate of the external CO.

*Fits at position of μCO (1810 cm^{-1}) and tCO (1968 cm^{-1}) modes of H_{ox}-CO might be affected by overlap with closely lying positive signals corresponding to the photoproduct H_{ox}.

**Growth time constant for the tCO mode of the photoproduct H_{ox}.
